# Activity of vehicles in the bus rapid transit system Metrobús in Mexico City

**DOI:** 10.1038/s41598-021-04037-6

**Published:** 2022-01-07

**Authors:** Jaspe U. Martínez-González, Alejandro P. Riascos

**Affiliations:** Instituto de Física, Universidad Nacional Autónoma de México, Ciudad Universitaria, 04510 Mexico City, Mexico

**Keywords:** Complex networks, Civil engineering

## Abstract

In this paper, we analyze a massive dataset with registers of the movement of vehicles in the bus rapid transit system Metrobús in Mexico City from February 2020 to April 2021. With these records and a division of the system into 214 geographical regions (segments), we characterize the vehicles’ activity through the statistical analysis of speeds in each zone. We use the Kullback–Leibler distance to compare the movement of vehicles in each segment and its evolution. The results for the dynamics in different zones are represented as a network where nodes define segments of the system Metrobús and edges describe similarity in the activity of vehicles. Community detection algorithms in this network allow the identification of patterns considering different levels of similarity in the distribution of speeds providing a framework for unsupervised classification of the movement of vehicles. The methods developed in this research are general and can be implemented to describe the activity of different transportation systems with detailed records of the movement of users or vehicles.

## Introduction

The study and understanding of human mobility in cities is an important and challenging problem since more than half of the world population lives in urban areas^1^. Nowadays human mobility can be explored in detail thanks to the digital traces people leave on mobile/digital platforms^2,3^. The identification of patterns in human mobility^4–9^ is necessary in topics like urban planning, dealing with traffic congestion^10^, the influence of the spatial distribution of a city^1,11–14^, the encounters or contacts that emerge^15,16^, among many others^1,2,17^. In these problems, the science of networks with well-established tools and methods to characterize and model complex systems^18–20^, provide a valuable framework to study transportation modes and their interactions^21–23^.

As one type of transit mode, bus rapid transit (BRT) systems have gained popularity worldwide for providing fast and easy access for citizens to fulfill their transportation needs^24^ and have been adopted widely over the world^24–27^. The merit of the BRT system lies in its ability to provide a high-quality public transit service with limited infrastructure and at relatively low capital and operating cost^25^. The benefits of a typical BRT system consist of dedicated lanes and proper vehicles and stations; such a layout guarantees a significant advantage in terms of operability^26^. In addition, BRT systems stand to significantly decrease personal vehicle mode share^25^ and might pull together connecting parts of the city in ways which other systems do not, especially at the level of service and spatial coherence^28^. In many BRT systems, vehicles have a preinstalled global positioning system (GPS) device which helps in collecting the travel time-related data, this information gives a global picture of the system in real time and can be used for improving the overall performance and schedule adherence of the vehicle. In recent works, the availability of trajectory data collected from operational vehicles in transportation systems has made possible the statistical analysis of travel time of vehicles in roadway segments^29–31^, the development of mathematical tools for the estimation of travel times and temporal changes in public transport^32,33^, the implementation of techniques to detect patterns in vehicle trajectories^34–36^ and public traffic congestion estimation by using artificial neural networks^37–41^. However, approaches to systematically analyze information and identify activity patterns in BRT systems are limited; specifically, very little past research in BRT systems focused on the statistical analysis of the speed of vehicles in specific zones of the system.

In contrast, community detection in networks^42–47^ has been proved as an important tool to detect patterns in different complex systems. For example, in the identification of correlations in financial markets^48,49^, the study of physiological networks^50^, the classification of patents based on their semantic content in technology^51^ and pharmaceutics^52^, describing the network of stations in bike-sharing systems^53,54^ or the geographical structure of the Twitter communication network at the global scale^55^, just to mention a few examples of the applicability of this method for pattern detection, unsupervised-classification, and data mining.

In this research, we analyze the activity of vehicles in the BRT system Metrobús in Mexico City. The database encompasses 383 days with registers of each active vehicle in the system with GPS geographical coordinates and speeds updated every 30 seconds. For this study, we divide the system into segments. In the first part, we applied statistical methods to characterize the movement of vehicles in each segment by comparing daily activity with the total data. Using the Kullback–Leibler distance between probability densities of speeds we identify zones with regular operation. In the second part, we compare the movement of vehicles in the system using a similarity network. In this structure, each segment is represented as a node, and links are added when the probability densities of speeds in two segments are similar. The exploration of different levels of similarity in terms of a parameter *H* define networks for which community detection algorithms allow an unsupervised classification of the segments based on the speeds of vehicles. The methods introduced are general and provide a framework for the study of different transportation systems in cities when massive databases with geolocalized activity are available. This approach will help to a better understanding of variations in the speeds over space and time by means of statistical analyses and complement other techniques such as time reliability-based performance indicators introduced to study the travel time variations of vehicles in specific routes.

## Results

### Global characteristics of the system

**Figure 1:**
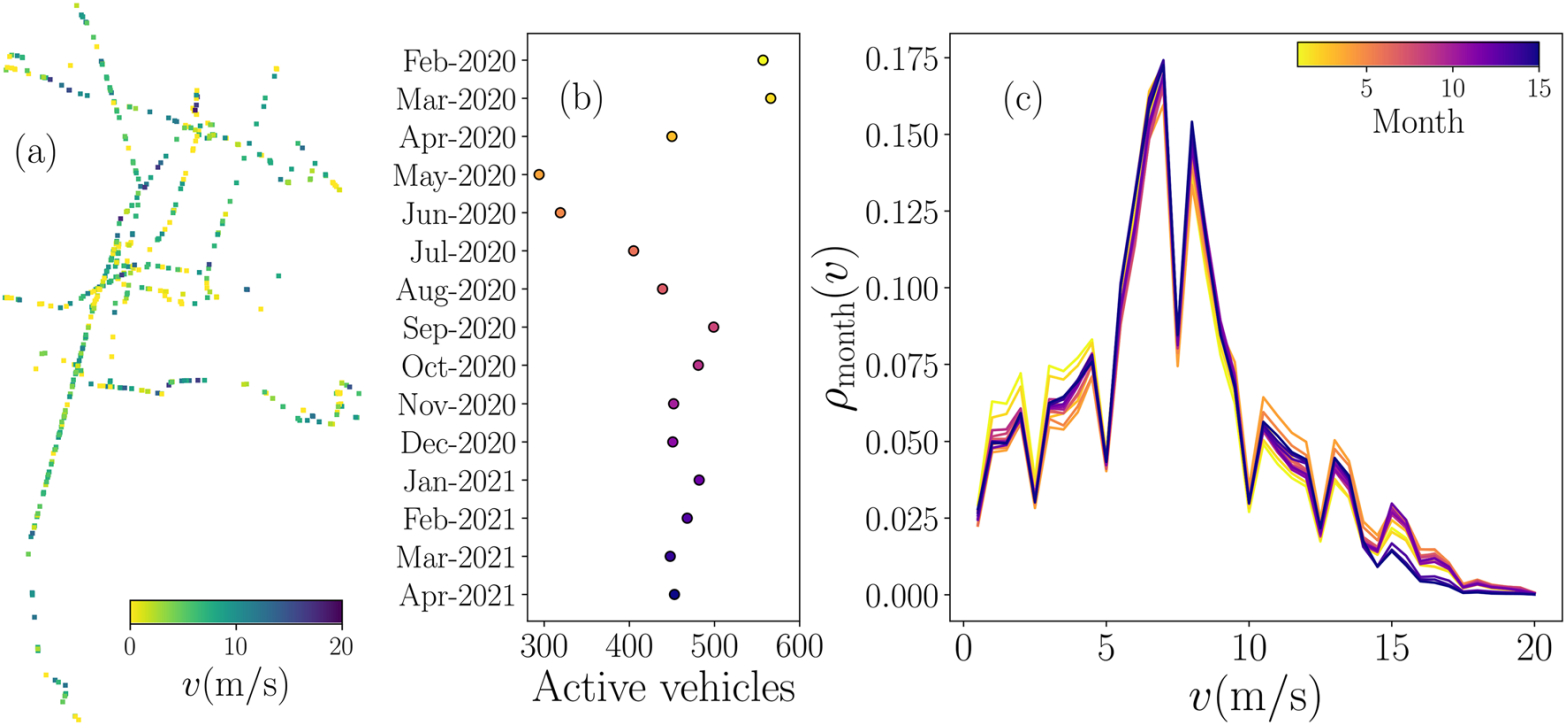
Global characteristics of the BRT system Metrobús in Mexico City. (**a**) Activity of vehicles at 13:00 h observed in registers on March 2nd, 2020. Each active vehicle in the system is represented by a point with speeds *v* encoded in the color bar. (**b**) Monthly active vehicles from February 2020 to April 2021. (**c**) Probability density $\rho _{\mathrm {month}}(v)$ of the speed *v* of active vehicles for each month in the color bar [the same colors are shown in the results in panel (**b**)]. We consider bin counts with increments $\Delta v=0.25\,{{\mathrm{m}}/{\mathrm{s}}}$. All figures were created using python 3.8 and the matplotlib (3.4.3) package (https://matplotlib.org).

The dataset analyzed in this research is part of an effort of Mexico City to have open databases for transportation systems, human mobility and other topics of interest^56^. In particular, for the BRT system Metrobús it is possible to have access to real-time registers with geographical coordinates (longitude, latitude) for positions and speeds of all the active vehicles in the system^57^. We analyze data for 383 days collected from February 2020 to April 2021 (see “Methods” section for a detailed description of the data). From these registers of activity we have the speed of each vehicle in ${{\mathrm{m}}/{\mathrm{s}}}$. In this way, we have access to a global picture of the system’s activity at specific moments.

In Fig. 1 we illustrate a global analysis of the dataset. In Fig. 1a we show the positions and speeds of the vehicles in March 2nd, 2020 at 13:00 h, the system has 516 active vehicles at this moment. Each vehicle is depicted with a point and the color encodes its speed (see Supplemental Material with a video of the complete data collected for this day). This representation gives a general overview of the data, such as regions with the highest activity, and zones where the speed of the units is higher than the average. On the other hand, considering that each vehicle has a unique ID, we can count the total number of active vehicles in the collected data on a determined time scale. In Fig. 1b, we show the number of active units at the scale of months considering the 15 months covered in this research. In this analysis, we assume that a vehicle is active in the system if at least has one register in the respective month. The results show that the number of vehicles changes significantly, especially due to the modifications in the system introduced in response to the different stages of the COVID-19 pandemic in Mexico City. In this respect, the number of vehicles in February and March of 2020 represents the common operation pre-COVID-19 in Mexico. This number reduced, particularly, from April to August 2020. In the last 8 months of this study, the number of active vehicles increased from the low in May 2020 but not to the same levels observed in the first two months. To complement this part, in Fig. 1c, we analyze the information of the speeds of the vehicles in the whole system in each month. We explore the probability density $\rho _{{\mathrm {month}}}(v)$ of non-null speeds *v* for the records of each month in the dataset. In strong contrast with the results for the number of vehicles, we see that the distributions of speeds of the vehicles present similar characteristics with small changes at lower speeds $v\le 5\,{{\mathrm{m}}/{\mathrm{s}}}$ and higher speeds $v\ge 10\,{{\mathrm{m}}/{\mathrm{s}}}$, minimal variations are observed for $5\,{{\mathrm{m}}/{\mathrm{s}}}<v< 10\,{{\mathrm{m}}/{\mathrm{s}}}$.

### Vehicle activity in segments

**Figure 2:**
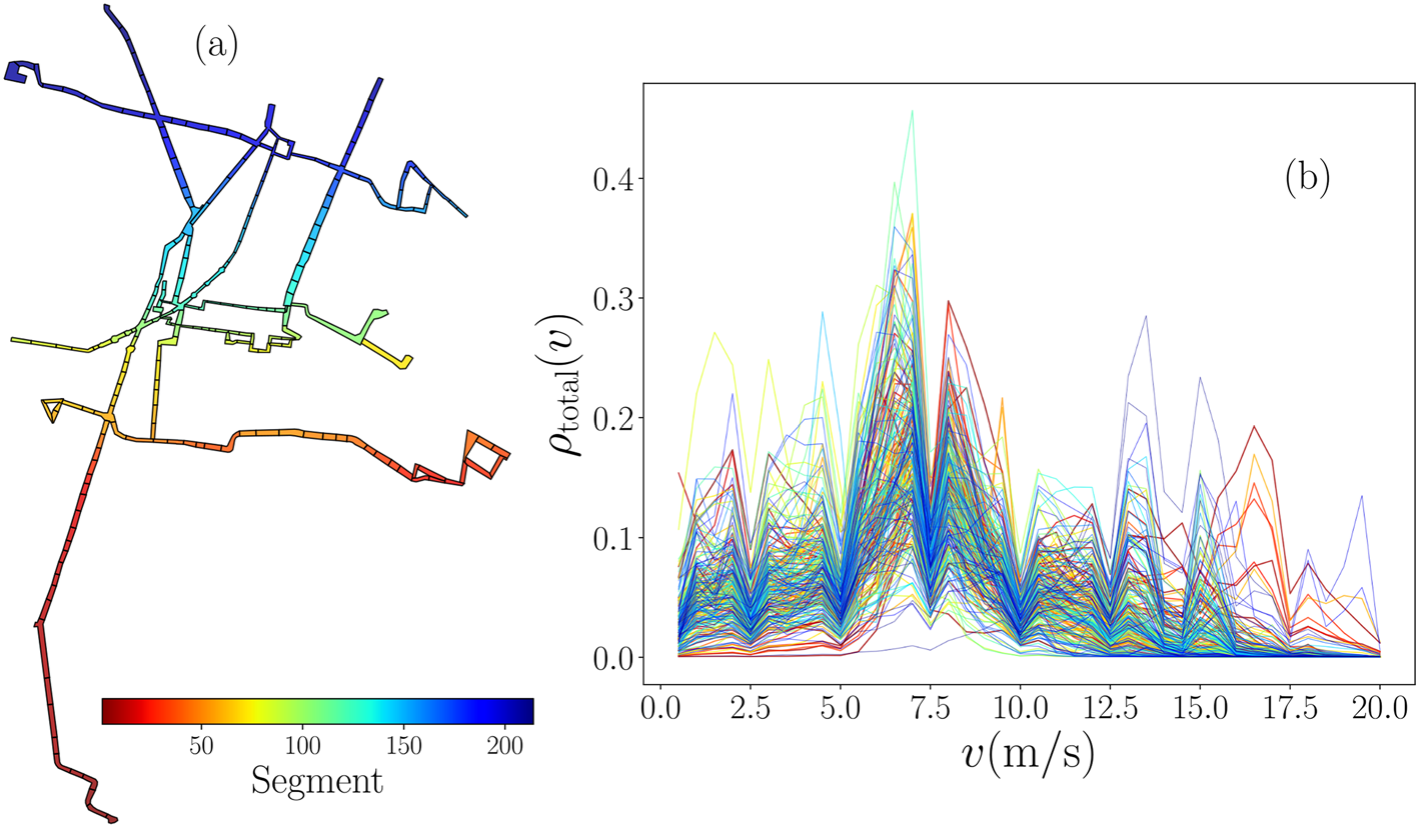
Activity of vehicles in segments of the system Metrobús in Mexico City. (**a**) Map with polygons associated to each of the ${\mathscr {N}}=214$ segments in the system. (**b**) Probability density $\rho _{{\mathrm {total}}}(v)$ of the speed *v* of vehicles in each segment. The values $\rho _{{\mathrm {total}}}(v)$ are obtained from bin counts in the interval $0<v\le 20\,{{\mathrm{m}}/{\mathrm{s}}}$ with increments $\Delta v=0.25\,{{\mathrm{m}}/{\mathrm{s}}}$. Segments are sorted considering their geographical coordinates with a number codified in the color bar that applies for polygons in (**a**) and the respective $\rho _{{\mathrm {total}}}(v)$ in (**b**). All figures were created using python 3.8 and the matplotlib (3.4.3) package (https://matplotlib.org). The map in panel (**a**) was created using the geopandas (0.10.2) package (https://geopandas.org).

In addition to the vehicles, the infrastructure of the system Metrobús includes 195 stations where users access this service and are distributed in 7 lines with 225 km exclusive roadways dedicated to buses. We use all the information available for the movement of vehicles to study the operation of the system in different zones of Mexico City. To this end, we divide the system into ${\mathscr {N}}=214$ segments defined by polygons that include stations and the lanes that connect them. A simple segment is described by an elongated rectangle defining a specific geographical region that includes the system’s roads and stations at two of its ends. In this study, our partition of the systems considers 205 simple segments. In addition, 9 segments are general polygons, located in zones where different lines converge. In Fig. 2a, we present all the segments of the system. In this representation, polygons are sorted according to the geographical coordinates of their geometrical centers starting from the south-west and considering the latitudes (from south to north) as the first variable and the longitudes (from west to east) as the second variable. An index $i= 1,2,\ldots ,{\mathscr {N}}$ codified in the color bar denotes the segment number; we maintain the same index in all the following analyses.

Once we define the information of polygons representing the segments, we proceed to analyze the activity of vehicles in each one of them. Due to the high volume of data, we use the geographical coordinates of the vehicles to divide the complete dataset into registers associated with each segment. In this manner, we have all the speeds in each segment during the 383 days in our study. The statistical analysis of the speeds *v* considering the movement of vehicles (i.e. only registers with $v>0\, {{\mathrm{m}}/{\mathrm{s}}}$ corresponding to $164\,867\,137$ speed values, a $76.7\%$ of the total database) are presented in Fig. 2b, results show the probability density $\rho _{{\mathrm {total}}}(v)$ calculated with the relative frequencies of *v* in regular bin counts with $\Delta v=0.25\,{{\mathrm{m}}/{\mathrm{s}}}$. In this representation, we maintain the same colors that codified the segments in Fig. 2a. We define a maximum speed $v_{{\mathrm {max}}}=20\,{{\mathrm{m}}/{\mathrm{s}}}=72\,{\mathrm {km/h}}$, only a $0.0114\%$ of the total database contains registers with $v>v_{{\mathrm {max}}}$. In addition, our analysis shows that in a high number of segments, most frequent values of speeds are observed in the interval $5\,{{\mathrm{m}}/{\mathrm{s}}}\le v\le 10\,{{\mathrm{m}}/{\mathrm{s}}}$.

Also, the values *v* can be divided into sets considering a defined time window; for example, registers in a particular hour, day, weekday, month, among others. Once we establish a particular partition of the speeds, we have probability densities that we can compare with the total density $\rho _{{\mathrm {total}}}(v)$ in each segment. On a temporal scale of days, we obtain 383 densities $\rho _{{\mathrm {day}}}(v)$ per segment with the information of the movement of vehicles in each day considered in our study. Then, using the Kullback–Leibler distance^58^

1 $$\begin{aligned} {{\mathscr {D}}}_{{\mathrm {KL}}}\equiv \int _0^{v_{\mathrm {max}}} \rho _{\mathrm {day}}(v)\log \left[ \frac{\rho _{\mathrm {day}}(v)}{\rho _{\mathrm {total}}(v)}\right] dv, \end{aligned}$$

we calculate the “*distance*” between each $\rho _{\mathrm {day}}(v)$ and $\rho _{\mathrm {total}}(v)$ (see the “Methods” section for a discussion about ${{\mathscr {D}}}_{{\mathrm {KL}}}$). In this way, we have 383 values of ${\mathscr {D}}_{\mathrm {KL}}$ in each segment comparing daily registers with the respective $\rho _{\mathrm {total}}(v)$ in Fig. 2b.

**Figure 3:**
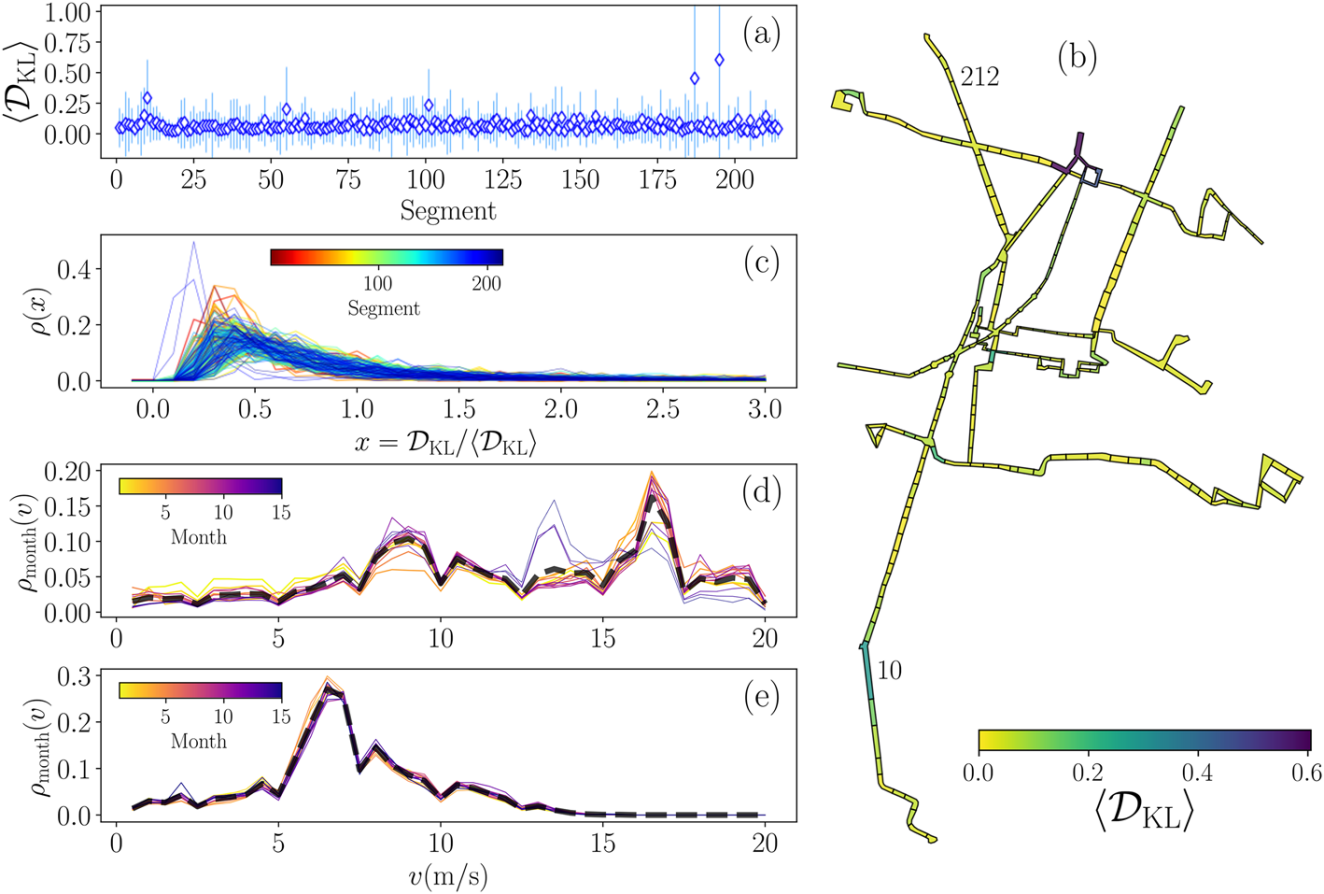
Statistical analysis of the distances ${{\mathscr {D}}}_{\mathrm {KL}}$ for speed densities. (**a**) Average Kullback–Leibler distances $\langle {\mathscr {D}}_{\mathrm {KL}}\rangle$ in each segment, error bars represent the standard deviation of the values. (**b**) Average values $\langle {\mathscr {D}}_{\mathrm {KL}}\rangle$ represented in the segments map. (**c**) Probability densities of the values ${\mathscr {D}}_{\mathrm {KL}}/\langle {\mathscr {D}}_{\mathrm {KL}}\rangle$ in each segment codified in the color bar. Probability densities $\rho _{{\mathrm {month}}}(v)$ of the speed *v* at the scale of months for: (**d**) segment 10 with $\langle {\mathscr {D}}_{\mathrm {KL}}\rangle =0.296$ and (**e**) segment 212 with $\langle {\mathscr {D}}_{\mathrm {KL}}\rangle =0.050$, dashed lines represent $\rho _{{\mathrm {total}}}(v)$ for the segment. All figures were created using python 3.8 and the matplotlib (3.4.3) package (https://matplotlib.org). The map in panel (**b**) was created using the geopandas (0.10.2) package (https://geopandas.org).

In Fig. 3 we present the statistical analysis of the distances ${\mathscr {D}}_{\mathrm {KL}}$ found. Figure 3a shows the average values $\langle {\mathscr {D}}_{\mathrm {KL}}\rangle$, error bars are obtained with the standard deviation of the values in each segment $\sigma _{\mathrm {KL}}=\sqrt{\langle {\mathscr {D}}_{{\mathrm {KL}}}^2\rangle -\langle {{\mathscr {D}}}_{{\mathrm {KL}}}\rangle ^2}$. We observe that the average values $\langle {\mathscr {D}}_{{\mathrm {KL}}}\rangle$ lie in the interval $0.0204\le \langle {\mathscr {D}}_{{\mathrm {KL}}}\rangle \le 0.6058$ and 209 segments present distances that can be considered as small with $\langle {\mathscr {D}}_{{\mathrm {KL}}}\rangle \le 0.155$. In contrast, in Fig. 3a we identify 5 segments $i=10,55,101,187,195$ with average distances $\langle {{\mathscr {D}}}_{{\mathrm {KL}}}\rangle > 0.2$. A detailed analysis of the Kullback–Leibler distances for these polygons reveal that in some days the distribution $\rho _{{\mathrm {day}}}(v)$ differs of the respective $\rho _{{\mathrm {total}}}(v)$, this may be due to modifications in the routes of vehicles. In Fig. 3b we plot the map of the system Metrobús, the colors represent the value $\langle {{\mathscr {D}}}_{{\mathrm {KL}}}\rangle$ of each segment. This map allows the identification of zones with regularity in the movement of vehicles (small values of $\langle {{\mathscr {D}}}_{{\mathrm {KL}}}\rangle$) and particular segments where average distances $\langle {{\mathscr {D}}}_{{\mathrm {KL}}}\rangle >0.2$ show that the daily distributions differ with the total activity captured in $\rho _{\mathrm {total}}(v)$. In Fig. 3c we depict the statistical analysis of the values $x={{\mathscr {D}}}_{\mathrm {KL}}/\langle {\mathscr {D}}_{\mathrm {KL}}\rangle$ in each segment, the respective probability densities $\rho (x)$ are skew with a high fraction of distances below the average, (i.e. with $x<1$).

In addition to the results for the ${\mathscr {D}}_{\mathrm {KL}}$ distances, we show in Fig. 3d,e the monthly distribution of speeds $\rho _{{\mathrm {month}}}(v)$ for two particular segments. Our analysis is similar to the presented for the total system in Fig. 1c, but now for the vehicles in segment 10 with $\langle {\mathscr {D}}_{\mathrm {KL}}\rangle =0.296$ and segment 212 with $\langle {\mathscr {D}}_{\mathrm {KL}}\rangle =0.050$ (the numbers of these particular segments are included in the map in Fig. 3b). With different colors, we represent the 15 months considered in our study. In the case of the segment 10 with higher distance $\langle {\mathscr {D}}_{\mathrm {KL}}\rangle$, the values of $\rho _{{\mathrm {month}}}(v)$ suffered deviations with respect with the total data $\rho _{{\mathrm {total}}}(v)$ represented with a dashed line. On the other hand, in the segment 212 with $\langle {{\mathscr {D}}}_{\mathrm {KL}}\rangle$ closer to zero, the $\rho _{{\mathrm {month}}}(v)$ remain approximately the same as $\rho _{{\mathrm {total}}}(v)$ for the total data in this segment.

The results in Fig. 3d,e are two particular examples to show how $\langle {\mathscr {D}}_{\mathrm {KL}}\rangle$ allows identifying variations in the activity of vehicles in a particular segment, similar analyses can be implemented at different scales of time to characterize changes or the regularity in the vehicular activity in a particular region of the system. In Fig. 3, it can be seen that except for the 5 segments discussed above, the activity of vehicles (described by $\rho _{{\mathrm {total}}}(v)$ in each segment) maintains some regularity. This result is important considering the variations in the number of active vehicles in Fig. 1b. A particular case is found in segment 10 that includes stations close to the National Autonomous University of Mexico. Since many of the activities in this university were developed virtually, the number of users in these stations was reduced significantly. As a consequence of the low demand, the movement of vehicles in this segment was restructured as we can see in Fig. 3d.

### Network of similarity between segments

Now we compare all the speed probability densities associated with the activity of vehicles in each segment. We consider the complete probability density for all segments $i=1,2,\ldots ,214$, with the records $0<v\le 20{{\mathrm{m}}/{\mathrm{s}}}$ in our database, denoted as $\rho ^{(i)}_{{\mathrm {total}}}(v)$ and presented in Fig. 2b. In this case, it is convenient using a symmetric distance ${\mathscr {D}}_{{\mathrm {KLS}}}(i,j)$ between two probability densities of the segments *i* and *j* obtained with the average of the Kullback–Leibler distance, hence we define

2 $$\begin{aligned} {\mathscr {D}}_{{\mathrm {KLS}}}(i,j)=\frac{{\mathscr {D}}_{\mathrm {KL}}(i,j)+{\mathscr {D}}_{{\mathrm {KL}}}(j,i)}{2} \end{aligned}$$

with

3 $$\begin{aligned} {{\mathscr {D}}}_{{\mathrm {KL}}}(i,j)\equiv \int _0^{v_{\mathrm {max}}} \rho ^{(i)}_{\mathrm {total}}(v)\log \left[ \frac{\rho ^{(i)}_{\mathrm {total}}(v)}{\rho ^{(j)}_{\mathrm {total}}(v)}\right] dv. \end{aligned}$$

**Figure 4:**
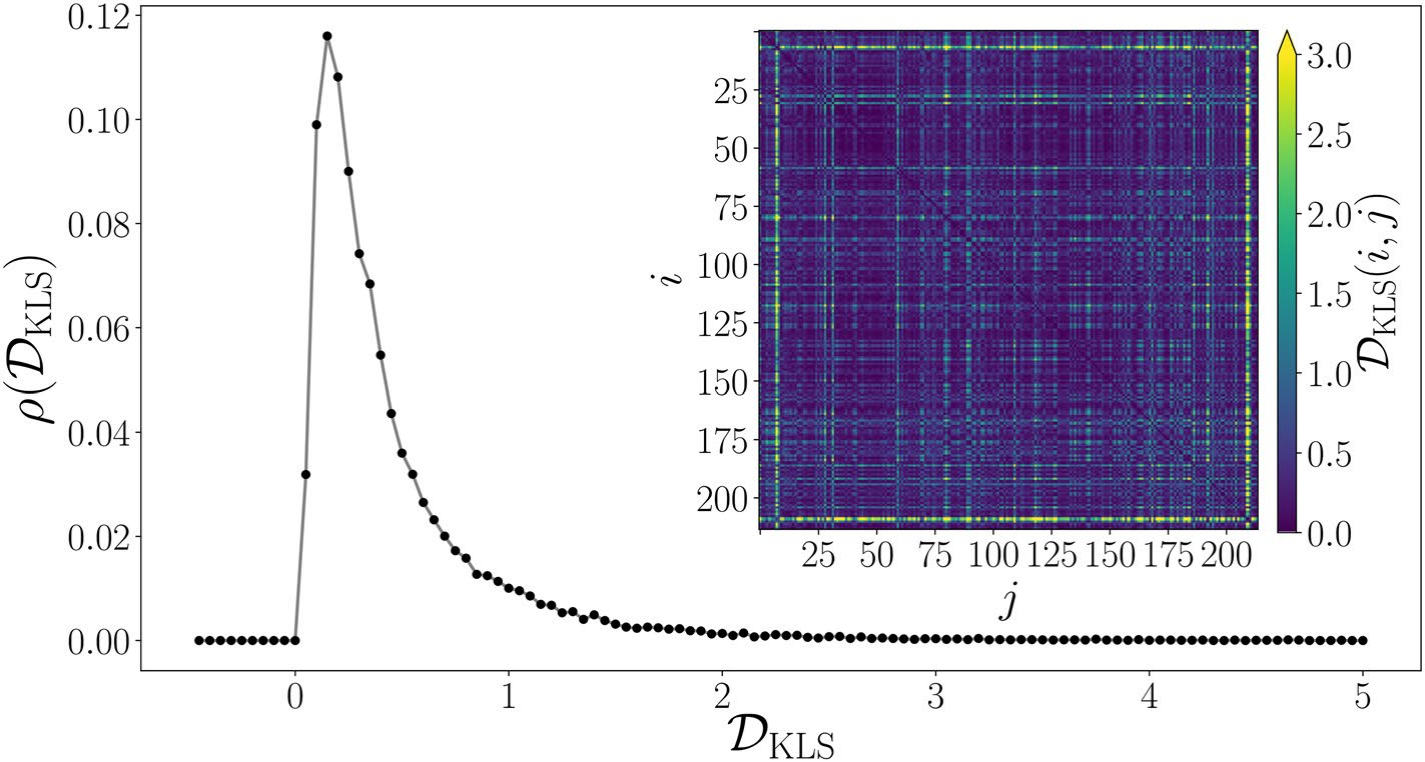
Statistical analysis of symmetric Kullback–Leibler distances between segments. The values ${\mathscr {D}}_{\mathrm {KLS}}$ are obtained from Eq. (2) for all the segment pairs $i,j=1,\ldots ,214$. We present the probability density $\rho ({\mathscr {D}}_{\mathrm {KLS}})$ obtained with bin counts in intervals with $\Delta {\mathscr {D}}_{\mathrm {KLS}}=0.05$, the inset shows the matrix representation with entries ${\mathscr {D}}_{\mathrm {KLS}}(i,j)$ codified in the color bar. All figures were created using python 3.8 and the matplotlib (3.4.3) package (https://matplotlib.org).

With the definition in Eq. (2), we obtain a ${\mathscr {N}}\times {\mathscr {N}}$ symmetric matrix with the information of similarity between the vehicle activity in the segments, the value ${\mathscr {D}}_{{\mathrm {KLS}}}=0$ is obtained for two equal speed distributions and values ${\mathscr {D}}_{{\mathrm {KLS}}}$ large show cases where the segments have a completely different activity. Our findings are depicted in Fig. 4, where we present as an inset the matrix with elements ${{\mathscr {D}}}_{{\mathrm {KLS}}}(i,j)$ for $i,j=1,\ldots ,214$. We also statistically analyze all the entries of the matrix of distances, obtaining the probability density $\rho ({{\mathscr {D}}}_{\mathrm {KLS}})$. Both representations show that a high fraction of the distances ${\mathscr {D}}_{\mathrm {KLS}}$ between segments have values in the interval $0\le {\mathscr {D}}_{\mathrm {KLS}}\le 0.5$, revealing different degrees of similarity in the activity in segments.

Looking for a better understanding of the similarities in all the distributions presented in Fig. 2b, we use the values of the distances in Fig. 4 to define a similarity network. In this representation, each node is associated with a segment of the BRT system, the size of the network is $N=214$ and an edge connecting two different nodes *i* and *j* is established if ${\mathscr {D}}_{{\mathrm {KLS}}}(i,j)\le H$ where *H* is a given threshold limit to decide if two segments have similar activity. The result is an undirected network with an adjacency matrix ${\mathbf {A}}$ with elements $A_{ij}=1$ if $0<{\mathscr {D}}_{{\mathrm {KLS}}}(i,j)\le H$ and $A_{ij}=0$ for ${\mathscr {D}}_{{\mathrm {KLS}}}(i,j)> H$. By definition, the adjacency matrix considers the diagonal entries $A_{ii}=0$ to avoid loops or connections of a node to itself. In the general case, it is hard to have intuition about the values of *H* to define a similarity network and its choice depends on the particular structure of the dataset explored and the metric used for the distance or relation between two nodes. In this respect, it is convenient to perform the statistical analysis of all the entries ${\mathscr {D}}_{\mathrm {KLS}}(i,j)$ presented in Fig. 4. In this representation of $\rho ({\mathscr {D}}_{\mathrm {KLS}})$, the area under the curve $\int _0^H\rho (z) dz$ gives the fraction of edges included in the network with respect to a fully connected graph. The higher the threshold *H*, the more edges are included in the network. In particular, in our analysis for the BRT system Metrobús, we see that the interval $0<H\le 1$ could produce networks with useful information. For $H\gg 1$, a high fraction of the edges are included in the similarity network losing any particular structure at the level of groups of zones. For the readers interested in this part, we refer to the recent work of Rincón et al.^52^, where a network of patents is explored using similar methods with a metric implemented to compare keywords in texts.

**Figure 5:**
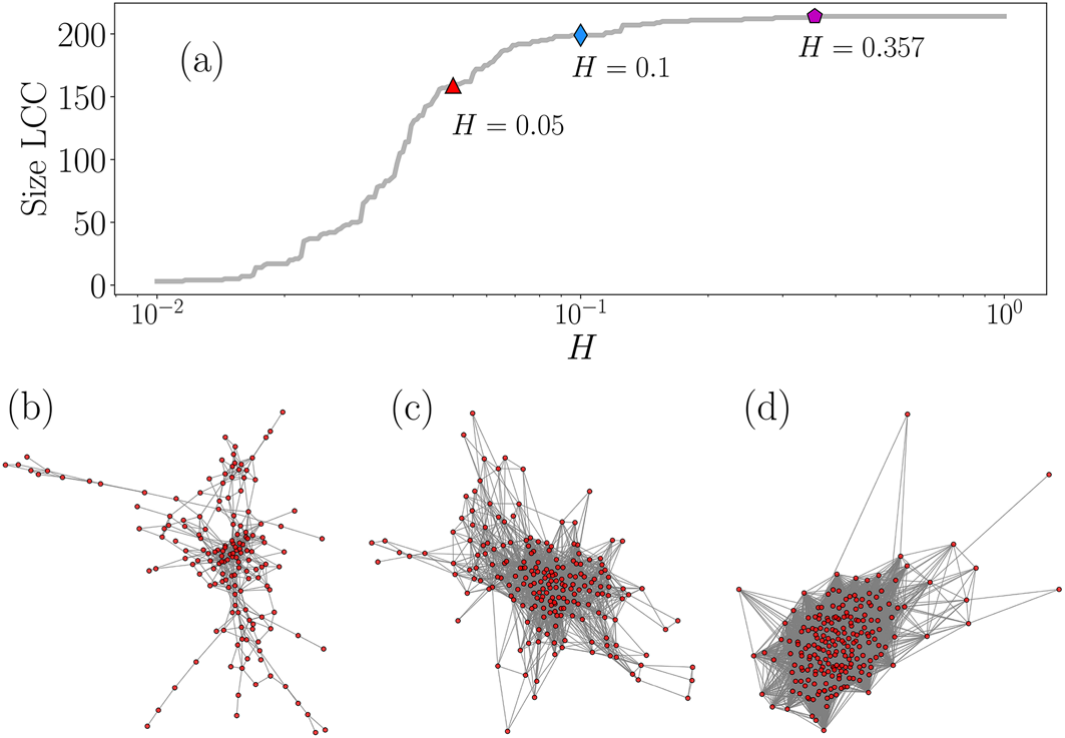
Similarity networks in the activity of vehicles in the BRT system Metrobús. (**a**) Size of the largest connected component (LCC) as a function of the threshold value *H* for the similarity between segments. Three particular LCCs obtained for $H=0.05$, $H=0.1$ and $H=0.357$ are presented in panels (**b**)–(**d**), respectively. $H=0.357$ is the lowest value of *H* that produces a connected network including the 214 segments. All figures were created using python 3.8 and the matplotlib (3.4.3) package (https://matplotlib.org). Networks in panels (**b**)–(**d**) were created using the networkx (2.6.3) package (https://networkx.org).

In this way, for each value of *H* we have a network of segments and we can apply standard methods for its analysis. In Fig. 5 we centered our study on the different networks generated and in the largest connected component (LCC), defined by the largest set of connected nodes within the network. In Fig. 5a we present the number of nodes in the LCC as a function of the similarity threshold *H* in the interval $0.01\le H \le 1$. In this representation of the results, we observe the effect of *H* defining different scales in the similarity between segments. For small values $H<0.02$ the similarity network is formed by small size disconnected clusters showing that a reduced number of probability densities are almost identical. This behavior changes in the interval $0.02\le H \le 0.1$ where the size of the LCC increases monotonically with *H*. For $H>0.1$ a high fraction of the network is connected and in $H\ge 0.357$ the LCC includes all the $N=214$ nodes. Here $H=0.357$ is the lowest value of *H* that produces a connected network including the 214 segments.

In this manner, all the information contained in the distance matrix can be analyzed considering different degrees of similarity of probability densities establishing connections between nodes as the structures in Fig. 5b–d obtained for the values $H=0.05$, $H=0.1$ and $H=0.357$. In the case with $H=0.05$, each edge requires high similarity between two segments and the LCC contains $N=159$ nodes, with an average degree $\langle k \rangle =7.7$, a global clustering coefficient $\left\langle C\right\rangle =0.410$ indicating that the structure has a low fraction of triangles; also, the average number of edges in the shortest path connecting two nodes in the network is $\langle l \rangle =4.42$ (see “Methods” section for a formal definition of $\langle k \rangle$, $\langle C \rangle$, $\langle l \rangle$ for networks with *N* nodes). In contrast, for $H=0.1$ the similarity network includes more edges defining a LCC with $N=199$ nodes, $\langle k \rangle =29.0$, $\left\langle C\right\rangle =0.598$ and $\langle l \rangle =2.64$ revealing a more connected structure. For $H=0.357$ the LCC contains all the $N=214$ nodes in a network with $\langle k \rangle =127$, $\left\langle C\right\rangle =0.822$ and $\langle l \rangle =1.48$ allowing a coarse-graining description of the segments in the system Metrobús. In networks with $H\gg 0.357$, increasing *H* we lose information of the similarity between segments and the network gradually approaches to a fully connected graph.

### Community structure and identification of patterns

**Figure 6:**
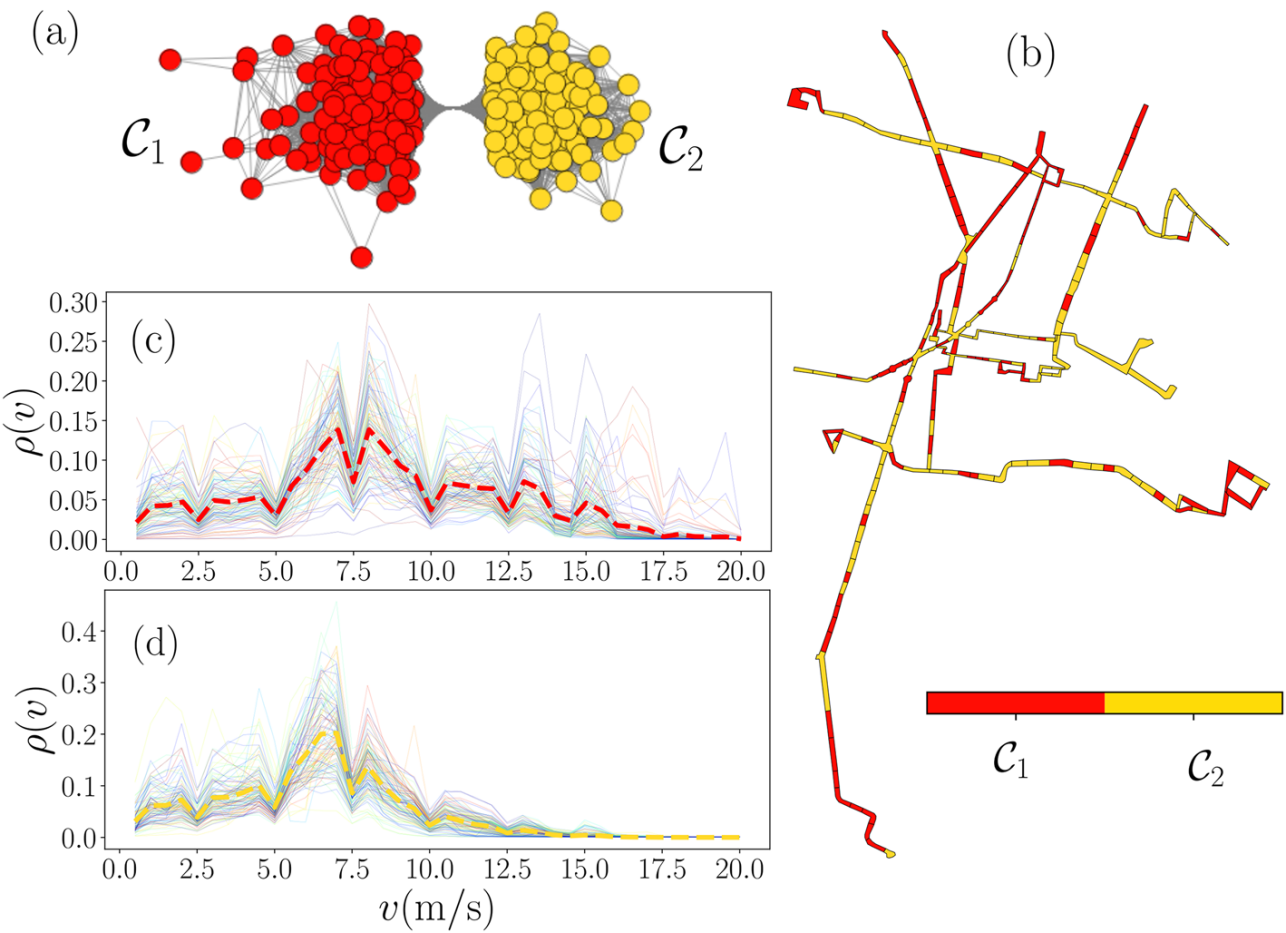
Community detection and activity of vehicles for similarity networks. (**a**) Similarity network with threshold value $H=0.357$ with two communities ${\mathscr {C}}_1$ and ${\mathscr {C}}_2$. (**b**) Segments map representing the communities. Probability densities $\rho (v)$ for the segments in ${\mathscr {C}}_1$ (**c**) and ${\mathscr {C}}_2$ (**d**). In panels (**c**) and (**d**), thin lines represent the $\rho ^{(i)}_{\mathrm {total}}(v)$ for each segment as in Fig. 2b and thick dashed lines depict the probability density found for all the values of *v* in $i\in {\mathscr {C}}_1$ and $i\in {\mathscr {C}}_2$. The network in panel (**a**) was generated using Mathematica 12.3.1 (https://www.wolfram.com/mathematica/). (**b**)–(**d**) were created using python 3.8 and the matplotlib (3.4.3) package (https://matplotlib.org). The map in panel (**b**) was created using the geopandas (0.10.2) package (https://geopandas.org).

**Figure 7:**
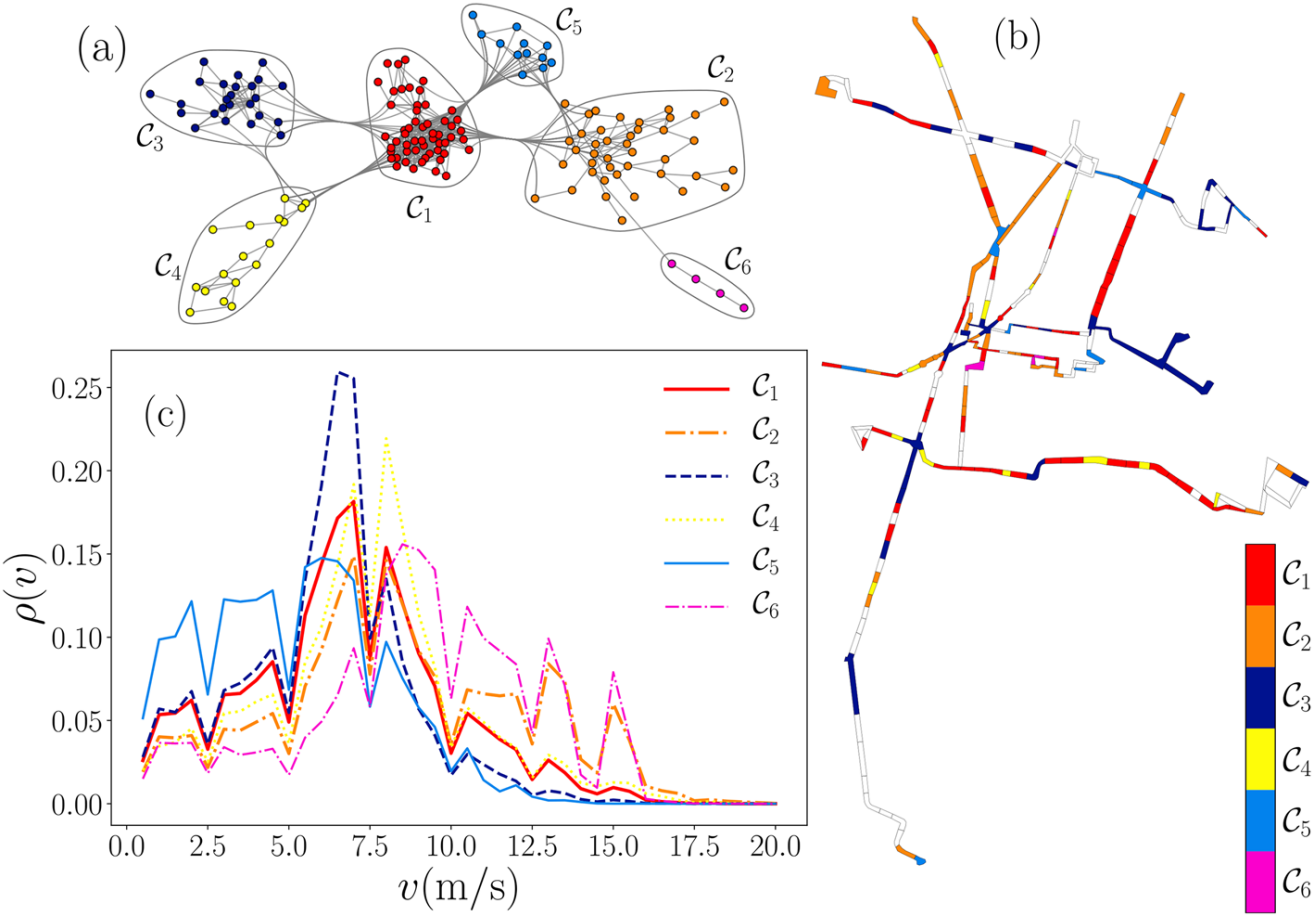
Segment classification through similarity networks with $H=0.05$. (**a**) Largest connected component for a similarity network with communities ${\mathscr {C}}_l$ ($l=1,2,\ldots ,6$) represented with different colors. (**b**) Segments map with the representation of communities. (**b**) Probability densities $\rho (v)$ for all the values in the segments in each community. The network in panel (**a**) was generated using Mathematica 12.3.1 (https://www.wolfram.com/mathematica/). Figures (**b**)–(**c**) were created using python 3.8 and the matplotlib (3.4.3) package (https://matplotlib.org). The map in panel (**b**) was created using the geopandas (0.10.2) package (https://geopandas.org).

In networks, the distribution of edges is not only globally, but also locally inhomogeneous, with high concentrations of edges within special groups of nodes and low connectivity between these groups. This feature in networks is called community structure. Communities (also clusters or modules), are sets of vertices that probably share common properties and/or play similar roles within the network^45^. Community detection endorses the identification of local connectivity patterns and guides the understanding of interactions in a complex structure. In this work, the communities represent groups of segments with similar activity of vehicles, which arose from considering all the information contained in the similarity network for different thresholds *H*, something not immediately visible if comparing the probability densities of speeds by pairs of segments. We apply modularity-based clustering algorithms^42,43^ to analyze the community structure of the LCC of similarity networks generated with $H=0.05$ and $H=0.357$ and depicted in Fig. 5b,d, the results derived from the community structure are presented in Figs. 6, 7 and Table 1.

**Table 1:** Classification of segments using community detection. Analysis of similarity networks for (a) $H=0.357$ and (b) $H=0.05$. The number of segments in each community is reported with the average degree $\langle k\rangle$, global clustering $\langle C\rangle$, and average lengths $\langle l \rangle$ of the shortest path connecting the nodes for each subnetwork. For the non-null speeds in the segments in each community ${\bar{v}}$ denotes the average speed and $\sigma _{v}$ the standard deviation, we also report the fraction (as percentage) of speeds with $v\ge 10\,{{\mathrm{m}}/{\mathrm{s}}}$.

Community	Segments	$\langle k\rangle$	$\langle C\rangle$	$\langle l\rangle$	${\bar{v}}({{\mathrm{m}}/{\mathrm{s}}})$	$\sigma _v({{\mathrm{m}}/{\mathrm{s}}})$	$\%$ $v\ge 10\,{{\mathrm{m}}/{\mathrm{s}}}$
**(a)** ${\varvec{H}}=\mathbf{0.357}$							
${\mathscr {C}}_1$	108	77.11	0.877	1.32	8.07	4.03	30.46
${\mathscr {C}}_2$	106	89.1	0.921	1.15	5.99	2.97	9.34
**(b)** ${\varvec{H}}=\mathbf{0.05}$							
${\mathscr {C}}_1$	55	11.7	0.625	2.33	6.41	3.17	13.28
${\mathscr {C}}_2$	41	3.85	0.287	3.36	8.05	3.81	30.69
${\mathscr {C}}_3$	32	4.75	0.494	2.83	5.71	2.61	5.31
${\mathscr {C}}_4$	12	2.67	0.281	2.41	6.93	3.09	14.2
${\mathscr {C}}_5$	10	3	0.250	1.91	4.73	2.69	3.16
${\mathscr {C}}_6$	4	1.5	0	1.67	8.68	3.57	37.81

In Fig. 6 we explore the Metrobús system using the similarity network with threshold limit $H=0.357$. This network has two communities: ${\mathscr {C}}_1$ with 108 nodes and ${\mathscr {C}}_2$ formed by 106 nodes; the community structure is represented in Fig.  6a, other quantities that characterize the subnetworks defining each cluster (average degree $\langle k\rangle$, global clustering $\langle C\rangle$ and average lengths of the shortest path connecting different nodes $\langle l \rangle$) are presented in Table 1(a). In Fig. 6b we show the communities in a map, in which segments in communities ${\mathscr {C}}_1$ and ${\mathscr {C}}_2$ are distributed in the whole system. We also present the total density of speeds $\rho ^{(i)}_{\mathrm {total}}(v)$ for the segments $i\in {\mathscr {C}}_1$ and $i\in {\mathscr {C}}_2$ (see panels in Fig. 6c,d), dashed lines are generated with the registers of *v* in all the segments representing the total activity in each community. The speed distributions found in the communities ${\mathscr {C}}_1$ and ${\mathscr {C}}_2$ present particular features. One of the differences observed is that in ${\mathscr {C}}_1$ the speeds $v\ge 10\,{{\mathrm{m}}/{\mathrm{s}}}$ appear in a $30.46 \%$ of the non-null records; whereas, only a $9.34 \%$ in ${\mathscr {C}}_2$ fulfill this condition. In this way, the classification of segments through community detection in similarity networks with $H = 0.357$ establishes a coarse-grained classification with two categories: ${\mathscr {C}}_1$ for high-speed segments and ${\mathscr {C}}_2$ for low-speed zones. The average speeds ${\bar{v}}$ in each community reported in Table 1(a) also confirm this characteristic observed in both categories, we also include the standard deviations $\sigma _v$ of the speed values. In the results in Fig. 6c,d, for $\rho ^{(i)}_{\mathrm {total}}(v)$ (represented with thin lines for each segment *i*), some probability densities deviate from the result obtained with the records for the whole community, something that is more marked in the ${\mathscr {C}}_1$ community. This is because having the threshold $H=0.357$, some differences are allowed in the similarity network.

In Fig. 7, we present the results for the analysis using $H=0.05$, a criterion that requires greater similarity to form a link in the network of segments. In this case, community detection algorithms applied to the LCC allow defining 6 communities ${\mathscr {C}}_1,{\mathscr {C}}_2,\ldots ,{\mathscr {C}}_6$ that contain at least four segments as we illustrate in Fig. 7a. In the map in Fig. 7b, we see that these categories produce a more varied map, although as *H* is small, several segments (represented in white) cannot be grouped into a community, being outside the LCC (we also omitted a community defined by three segments in the LCC). In Fig. 7c we present the probability density for *v* with the records of non-null speeds in the segments of each community. In this case, the unsupervised classification of the segments produces more varied results that are reported in Table 1(b). For example, in the analysis of the proportion in which the velocities $v\ge 10\,{{\mathrm{m}}/{\mathrm{s}}}$ appear, ${\mathscr {C}}_2$, ${\mathscr {C}}_6$ define groups of segments with high speeds where more than $30\%$ of the data meet $v\ge 10\,{{\mathrm{m}}/{\mathrm{s}}}$. Communities ${\mathscr {C}}_1$, ${\mathscr {C}}_4$ have a fraction of around $14\%$ for these speeds, and ${\mathscr {C}}_3$, ${\mathscr {C}}_5$ define low-speed zones with less than $10\%$. This type of classification is also evident for the average velocities ${\bar{v}}$ in each community, being the highest ${\bar{v}}= 8.68\,{{\mathrm{m}}/{\mathrm{s}}}$ in community ${\mathscr {C}}_6$ and the lowest ${\bar{v}}= 4.73\,{{\mathrm{m}}/{\mathrm{s}}}$ obtained for ${\mathscr {C}}_5$. The measures that describe the communities as networks also give us important information; for example, ${\mathscr {C}}_1$ and ${\mathscr {C}}_2$ are the subnetworks that have more nodes; however, considering the links, ${\mathscr {C}}_1$ is much more connected, a fact that is evidenced in the highest average degree and clustering. Finally, the most valuable information is the probability density $\rho (v)$ in Fig. 7c for the data in each community. The obtained distributions have particular characteristics that describe the vehicular movement in each group of segments. All this information and the map in Fig. 7b, help us to understand the global activity of the Metrobús system and its operation since the results obtained with the combination of methods implemented in this research allow us to detect emerging patterns when comparing the activity of the entire system.

## Discussion

From the study of the data with the activity of vehicles in the BRT system Metrobús for 383 days and a partition of the regions where these vehicles move defining 214 segments, it is found that this system operates with relative regularity in each zone. In particular, the distributions of speeds in the entire system and in each of the segments are preserved, presenting small variations depending on the day, with some exceptions that also can be detected using the statistical methods implemented for this study. In this way, the speed distribution of each segment is a good reference for the specific behavior of the vehicles in each of the geographical zones defining the segments. The variations in each segment at different temporal scales can be effectively studied using Kullback–Leibler distances.

In addition, the analysis of the Kullback–Leibler distance between speed distributions of all pairs of segments allows the representation of the entire system as a network. In this structure, community detection algorithms allow identifying groups of segments with similar vehicular activity, the number of communities found varies according to parameter *H* required to define the similarity network. In a case with $H=0.357$ two categories are established, one with segments in which speeds with $v \ge 10 \, {{\mathrm{m}}/{\mathrm{s}}}$ are more frequent and another in which these speeds appear less frequently. The analysis of a network with a greater similarity between segments with $H = 0.05$ gives a classification with more specific characteristics in the speed distributions, in this case the records with $v \ge 10 \, {{\mathrm{m}}/{\mathrm{s}}}$ appear in different proportions in each community. Our findings show that the Metrobús BRT system presents certain regularity in its operation, in the sense that the distribution of speeds of vehicles in 209 segments of the system suffered only small variations even with the reduction of active vehicles implemented due to the COVID-19 pandemic in Mexico City. It seems plausible to associate this regularity with the exclusive lanes in the system and the rules that operators of the vehicles must follow.

The statistical methods and the network science approach implemented in this research can be used for the multi-scale study of different transportation systems. In systems like taxis, buses, car-sharing services, in which large amounts of data are available with registers of the movement or quantities associated with vehicles or agents along with their geographic coordinates, this approach can lead to the unsupervised detection of regions with similar activity of vehicles. Other studies can incorporate the statistical analysis of different quantities of interest; for example, schedule adherence of the vehicles, carbon emissions, or user’s accessibility to stations. A profound understanding of the vehicle activity and similarities detected in groups of segments can help researchers and transit specialists to draw up strategies tailored to improving operational aspects of the system.

## Methods

### Dataset description

With the implementation of location-enabled devices on public transportation, a large amount of bus trajectory data is being generated. Since April 2019 all the information of the movement of all active vehicles in the BRT system Metrobús is available under request to the public^57^. The information provided contains the timestamp, vehicles ID, and registers in real-time of the GPS coordinates (longitude, latitude) of each vehicle in the system, their speeds in meters per second $({\mathrm {m}}/{\mathrm {s}})$, and qualitative descriptions of the state of each vehicle or the levels of congestion for example: *on time*, *stopped*, among others. Each vehicle updates this information every 30 seconds. The Metrobús system operates from 4:30 to 00:00 h on weekdays (Monday to Friday) and starts operation at 5:00 h on weekends (Saturday, Sunday) and holidays, the description of stations and routes is available to the public in the webpage of Metrobús^59^. By using a code written in Python, we request the data automatically (waiting 30 seconds between requests), an initial treatment of the retrieved records is performed to save the data. We maintained the download of data from February 16th, 2020 to April 8th, 2021. In total, we have data for 383 days with $215\,025\,258$ registers of position and speed of vehicles. On some particular days, the data was not available for download due to maintenance or problems connecting with the server.

### Kullback–Leibler distance

The Kullback–Leibler distance is a standard method to calculate the difference between two probability distributions *P*(*z*) and *Q*(*z*) describing a stochastic variable *z*^58,60^. This tool is widely used for database comparison. For continuous distributions, this distance is given by^58^

4 $$\begin{aligned} {\mathscr {D}}_{{\mathrm {KL}}}(P||Q) = \int P(z)\log \left[ \frac{P(z)}{Q(z)}\right] dz. \end{aligned}$$

Here *Q* acts as a reference distribution. Also, it is important to emphasize that ${\mathscr {D}}_{{\mathrm {KL}}}(P||Q)$ is not a distance in the sense of a metric since the distance between *P* and *Q* is not necessarily the same as between *Q* and *P*. Also, from the definition in Eq. (4), it is clear that ${\mathscr {D}}_{{\mathrm {KL}}}(P||Q)>0$ and is null when $P=Q$.

### Networks

Symmetric networks with *N* nodes are described by an adjacency $N\times N$ adjacency matrix ${\mathbf {A}}$ with entries 1 if two different nodes are connected and 0 otherwise. An important quantity in the study of networks is the degree of node *i* given by $k_{i}=\sum _{l=1}^N A_{il}$, that gives the number of connections to that node. In terms of this quantity we define the average degree as

5 $$\begin{aligned} \left\langle k\right\rangle =\frac{1}{N}\sum _{i=1}^N k_i. \end{aligned}$$

Another measure to characterize the topology of networks is the clustering coefficient^18^. This coefficient $C_i$ of the node *i*, quantifies the fraction of connected neighbors ${\triangle }_i$ of the node *i* with respect to the maximum number of these connections given by $k_i(k_i-1)/2$. In terms of the adjacency matrix we have for $k_i\ge 2$

6 $$\begin{aligned} C_i=\frac{ ({{\mathbf {A}}}^3)_{ii}}{k_i(k_i-1)}, \end{aligned}$$

otherwise $C_i=0$. Here $({\mathbf {A}}^3)_{ii}=({\mathbf {A}}{\mathbf {A}}{\mathbf {A}})_{ii}={\triangle }_i/2$. The average clustering coefficient is given by

7 $$\begin{aligned} \left\langle C\right\rangle =\frac{1}{N}\sum _{i=1}^N C_i. \end{aligned}$$

From the information in the adjacency matrix, different algorithms allow the calculation of the shortest path connecting the nodes *i* and *j*, the length $l_ {ij}$ with the number of edges in this shortest path is a measure of the distance between two nodes^18^. This information allows defining an average distance $\langle l\rangle$ given by

8 $$\begin{aligned} \langle l\rangle =\frac{1}{N(N-1)}\sum _{i=1}^N\sum _{j=1}^N l_{ij}. \end{aligned}$$

In this way, for a particular connected undirected network with *N* nodes, we can calculate the adjacency matrix ${\mathbf {A}}$ and obtain the global quantities $\left\langle k \right\rangle$, $\left\langle C \right\rangle$ and $\langle l\rangle$ that describe this structure.

## Supplementary Information

Supplementary Video 1.

Supplementary Legends.
